# Colonoscopy compliance and diagnostic yield in a large population-based colorectal cancer screening programme

**DOI:** 10.1007/s00384-023-04517-3

**Published:** 2023-09-13

**Authors:** Xinzhu Zhao, Shuyuan Wang, Zhen Yuan, Suying Yan, Wenwen Pang, Xinyu Liu, Wanting Wang, Ben Yi, Qiurong Han, Qinghuai Zhang, Xipeng Zhang, Chunze Zhang

**Affiliations:** 1 Department of Colorectal Surgery, Tianjin Union Medical Center, Tianjin, China; 2 School of Medicine, Nankai University, Tianjin, China; 3 School of Integrative Medicine, Tianjin University of Traditional Chinese Medicine, Tianjin, China; 4 Tianjin Medical University, Tianjin, China; 5 Department of Clinical Laboratory, Tianjin Union Medical Center, Tianjin, China; 6 The Institute of Translational Medicine, Tianjin Union Medical Center, Tianjin, China; 7 Tianjin Institute of Coloproctology, Tianjin, China

**Keywords:** Colorectal cancer (CRC), colonoscopy, Compliance, Advanced adenomas (AA)

## Abstract

**Objectives:**

With the intention of providing a reference for secondary prevention, our study provides some insight on diagnostic yield of factors influencing compliance with colonoscopy and the presence of advanced adenomas (AA).

**Methods:**

We conducted large-scale CRC screening among local Tianjin residents aged 40–75 years between 2012 and 2019. A high-risk factor questionnaire (HRFQ) was distributed to each participant, followed by the performance of a fecal immunochemical test (FIT). Participants who tested positively for any of these items were advised to undergo a colonoscopy. Relevant basic information was collected from participants during CRC screening, and the screening data were sorted and analysed.

**Results:**

A total of 5,670,924 people participated in CRC screening by the end of 2019, including 275,708 people in the high-risk group, and 74,685 (27.1%) people who underwent colonoscopy. The results of the logistic regression model demonstrated that participants with a history of mucous bloody stool (OR = 8.20, 95% CI: 7.92, 8.50, p < 0.001), chronic diarrhea (OR = 5.73, 95% CI: 5.57, 5.89, p < 0.001), and higher level of education (OR = 1.87, 95% CI: 1.80, 1.93, p < 0.001) were more likely to comply with a colonoscopy. Several factors including age (70–75 years old:OR = 3.72, 95% CI: 2.71, 5.10, p < 0.001), and FIT( +) (OR = 1.65, 95% CI: 1.42,1.90, p < 0.001) were identified to be associated with the presence of AA.

**Conclusions:**

Increased compliance with colonoscopy is urgently needed. Our findings can inform the design of future effective large-scale population-based CRC screening programmes.

**Supplementary Information:**

The online version contains supplementary material available at 10.1007/s00384-023-04517-3.

## Introduction

Globally, colorectal cancer (CRC) is the third-most prevalent and second-most deadly cancer [1]. It is estimated that the global burden of CRC will increase by 60% by 2030, with more than 2.2 million new cases and 1.1 million deaths [2]. Countries with a developed economy have a higher incidence of CRC, yet it is progressively rising in developing nations due to all aspects of westernization [3]. China has experienced an increasing trend in CRC incidence and mortality over the last few decades [4]. A contributing factor to this trend is the lack of early detection and treatment of CRC [5].

CRC may be able to be prevented via screening due to its long timeline from precancerous (polyps and AA) to cancerous phase and probably have an optimistic prognosis [6, 7]. In randomized controlled trials as well as observational studies, early detection of lesions through different screening tools substantially reduces disease burden [8, 9]. Researchers have found that FIT may lower CRC incidence and mortality rates by 10% and 22%-62% [10–12]. Colonoscopy reduces rates of incidence and mortality by about 31%-69% and 29%-67%, [13–15] respectively, which is considerably higher than FIT. For CRC screening, colonoscopy is regarded as the gold standard, reducing CRC-specific mortality by 68% [16]. A significant reduction in both distal and proximal CRC was associated with colonoscopy [17]. Colonoscopy plays an essential role in the overall screening procedure for CRC. Promoting and encouraging people to participate colonoscopy could be the aim of public health department.

Unfortunately, although CRC screening has long been available in various countries, the compliance of screening programmes utilizing colonoscopy as the major method were disappointing [18], which may considerably reduce the efficiency of CRC screening by lowering the actual CRC or adenoma detection rate [19]. Our study describes the results of CRC screening in Tianjin from 2012 through 2019 and focuses on compliance of colonoscopy and AA.

## Methods

### Study design and study population

This study involved data from the 2012–2019 CRC screening programme conducted in Tianjin. CRC screening was performed with Tianjin Union Medical Center as the technical support unit. The participating screening units included more than 300 community hospitals in 16 districts and counties. Following informed consent, each participant completed the HRFQ and was required to undergo a FIT, positive results in either or both items were considered to indicate high CRC risk. If there were no contraindications to colonoscopy, the high-risk individuals would be recommended to go through a colonoscopy, along with biopsy and/or polypectomy, if necessary. The pathological findings of the colonoscopy and other detailed information of these participants were analyzed in depth.

The HRFQ includes items that assess demographic characteristics as well as CRC-related risk factors. A positive result on the HRFQ (HRFQ +) results from one or more of the following:

- (1) A first-degree relative with CRC or (2) a history of cancer or polyps; and/or two or more of the following:
  - (a) Chronic constipation, (b) chronic diarrhea, (c) mucous-bloody stool, (d) a history of adverse life events, (e) a history of chronic appendicitis or appendectomy, or (f) a history of gallbladder disease or gallbladder surgery.

Remarks: (1) Chronic diarrhea refers to diarrhea cumulatively lasting for more than 3 months in the last 2 years, with each episode lasting more than 1 week; (2) chronic constipation refers to constipation for more than 2 months per year in the last 2 years; (3) adverse life events must have occurred within the last 20 years and caused significant mental trauma or distress to the individual after the event (e.g., divorce, loss of spouse, or loss of child). [20].

In addition, the HRFQ also collected information on sex, age, height, weight, smoking status, alcohol intake, exercise frequency, marital status, occupation, and educational background of participants to establish a foundation for more detailed analysis of factors linked to colorectal tumours. In the FIT, each participant provided a 10–50-mg stool sample without dietary restriction. Individuals were instructed to send this sample to the appropriate screening hospital laboratory on the same day. Analysis of samples occurred no later than 8 h after participant collection.

**Fig. 1:**
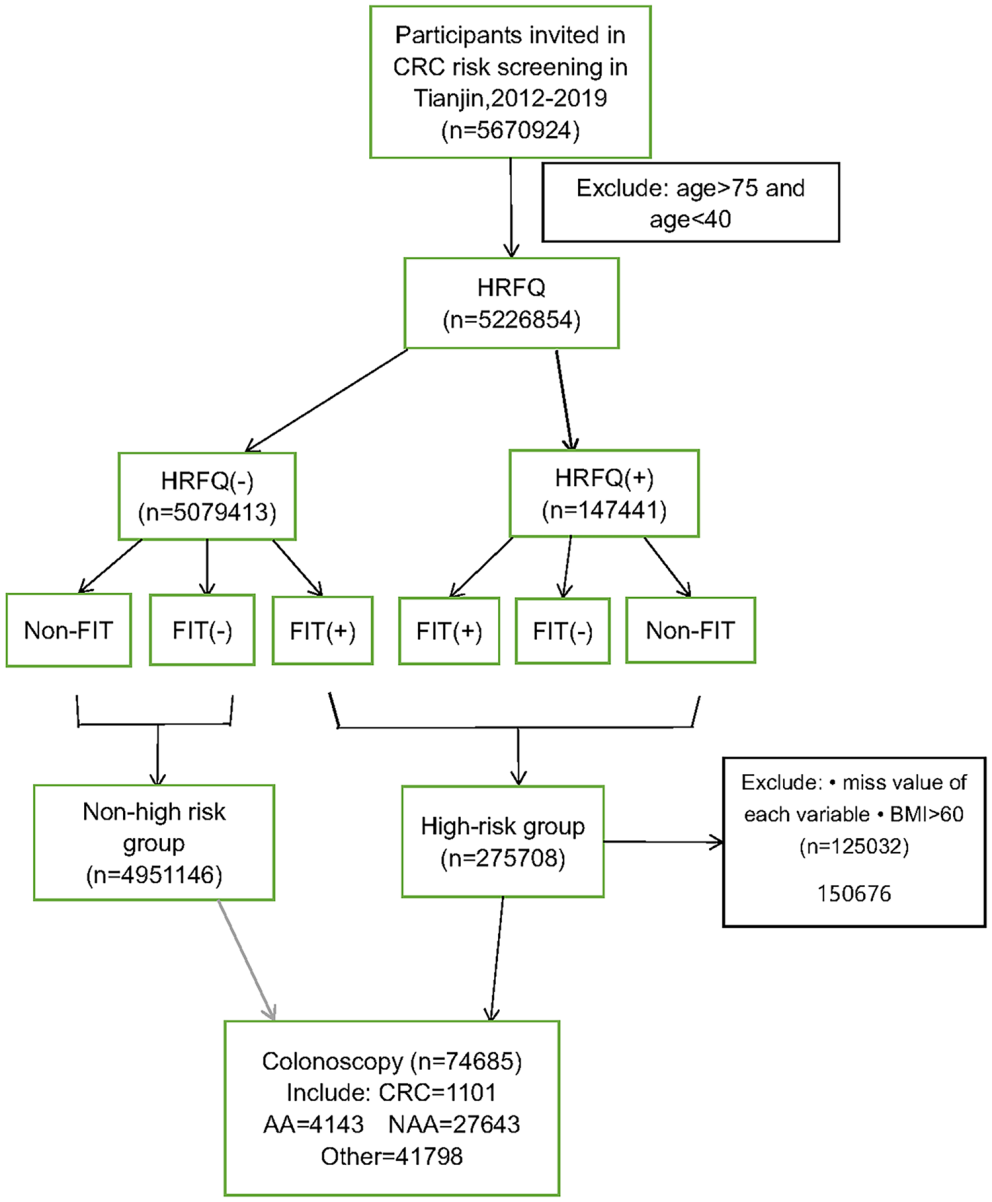
Flow chart of the Tianjin CRC screening programme, 2012–2019 FIT: fecal immunological test; HRFQ: high-risk factor questionnaire; CRC: colorectal cancer; HRFQ( +): positive result on the HRFQ; FIT( +): positive result on the FIT; HRFQ(-): negative result on the HRFQ; FIT(-): negative result on the FIT

Figure 1 shows that 5,670,924 participants were recruited for the project. After restricting the age of participants to 40–75 years, we retained 5,226,854 participants and identified 275,708 (5.27%) participants from them with high risk of CRC according to the screening. In the end, 74,685 (27.1%) people complied with the colonoscopy. We excluded those participants who missed important variables needed to explore the association with colonoscopy adherence (e.g., marital status, educational background, smoking status, alcohol intake, exercise frequency, BMI) from the high-risk group(n = 275,708), retaining 150,676 participants included in Table 1.

**Table 1:** Sample characteristics stratified by complied with colonoscopy or not

**Factors**	**Level**	**Overall**	**Colonoscopy** **( +) (%)**	**Colonoscopy** **(-) (%)**		***p***
		150,676	32,762(21.7)	117,914(78.3%)		
**Sex**	Women	88,230 (58.6)	17,477 (53.3)	70,753 (60.0)	< 0.001	
	Men	62,446 (41.4)	15,285 (46.7)	47,161 (40.0)		
**Age(years)**	40–49	6988 (4.6)	1527 (4.7)	5461 (4.6)	< 0.001	
	50–59	37,245 (24.7)	8125 (24.8)	29,120 (24.7)		
	60–69	70,516 (46.8)	16,855 (51.4)	53,661 (45.5)		
	70–75	35,927 (23.8)	6255 (19.1)	29,672 (25.2)		
**Marital status**	Married	128,005 (85.0)	31,289 (95.5)	96,716 (82.0)	< 0.001	
	Unmarried	1270 (0.8)	288 (0.9)	982 (0.8)		
	Divorced	3823 (2.5)	270 (0.8)	3553 (3.0)		
	Widowed	17,578 (11.7)	915 (2.8)	16,663 (14.1)		
**Educational background**	Low	42,120 (28.0)	6996 (21.4)	35,124 (29.8)	< 0.001	
	Intermediate	94,627 (62.8)	21,814 (66.6)	72,813 (61.8)		
	High	13,929 (9.2)	3952 (12.1)	9977 (8.5)		
**Job**					< 0.001	
	Office staff	6540 (4.3)	1589 (4.9)	4951 (4.2)		
	Others	9940 (6.6)	2420 (7.4)	7520 (6.4)		
	Director	15,536 (10.3)	3120 (9.5)	12,416 (10.5)		
	Soldier	79 (0.1)	21 (0.1)	58 (0.0)		
	Production staff	40,696 (27.0)	8158 (24.9)	32,538 (27.6)		
	Business and service personnel	6316 (4.2)	1477 (4.5)	4839 (4.1)		
	Equipment operator	27,599 (18.3)	6378 (19.5)	21,221 (18.0)		
	Jobless	25,821 (17.1)	5114 (15.6)	20,707 (17.6)		
	Professional technician	18,116 (12.0)	4483 (13.7)	13,633 (11.6)		
**Smoking status**	Never	112,005 (74.3)	24,871 (75.9)	87,134 (73.9)	< 0.001	
	Past	6685 (4.4)	1527 (4.7)	5158 (4.4)		
	Present	31,986 (21.2)	6364 (19.4)	25,622 (21.7)		
**Alcohol intake**	Never/occasional	135,041 (89.6)	29,181 (89.1)	105,860 (89.8)	< 0.001	
	Weekly/daily	15,635 (10.4)	3581 (10.9)	12,054 (10.2)		
**Exercise frequency**	< 1time/week	85,550 (56.8)	18,136 (55.4)	67,414 (57.2)	< 0.001	
	> 1time/week	65,126 (43.2)	14,626 (44.6)	50,500 (42.8)		
**BMI**	< 25	82,569 (54.8)	18,332 (56.0)	64,237 (54.5)	< 0.001	
	25–30	56,447 (37.5)	12,192 (37.2)	44,255 (37.5)		
	> 30	11,660 (7.7)	2238 (6.8)	9422 (8.0)		
**History of chronic diarrhea**	No	120,877 (80.2)	25,037 (76.4)	95,840 (81.3)	< 0.001	
	Yes	29,799 (19.8)	7725 (23.6)	22,074 (18.7)		
**History of chronic constipation**	No	109,662 (72.8)	25,052 (76.5)	84,610 (71.8)	< 0.001	
	Yes	41,014 (27.2)	7710 (23.5)	33,304 (28.2)		
**History of mucous bloody stool**	No	132,974 (88.3)	27,038 (82.5)	105,936 (89.8)	< 0.001	
	Yes	17,702 (11.7)	5724 (17.5)	11,978 (10.2)		
**History of appendicitis or appendectomy**	No	131,119 (87.0)	29,860 (91.1)	101,259 (85.9)	< 0.001	
	Yes	19,557 (13.0)	2902 (8.9)	16,655 (14.1)		
**History of gallbladder disease or gallbladder surgery**	No	132,551 (88.0)	29,972 (91.5)	102,579 (87.0)	< 0.001	
	Yes	18,125 (12.0)	2790 (8.5)	15,335 (13.0)		
**History of adverse life events**	No	120,253 (79.8)	29,779 (90.9)	90,474 (76.7)	< 0.001	
	Yes	30,423 (20.2)	2983 (9.1)	27,440 (23.3)		
**History of cancer**	No	140,105 (93.0)	31,347 (95.7)	108,758 (92.2)	< 0.001	
	Yes	10,570 (7.0)	1415 (4.3)	9155 (7.8)		
**Previously detected colonic polyp**	No	139,676 (92.7)	29,243 (89.3)	110,433 (93.7)	< 0.001	
	Yes	11,000 (7.3)	3519 (10.7)	7481 (6.3)		
**History of CRC in first-degree relatives**	No	133,839 (88.8)	29,359 (89.6)	104,480 (88.6)	< 0.001	
	Yes	16,457 (10.9)	3349 (10.2)	13,108 (11.1)		
	Unknown	380 (0.3)	54 (0.2)	326 (0.3)		
**Past fecal immunochemical test**	No	20,643 (13.7)	3986 (12.2)	16,657 (14.1)	< 0.001	
	Yes(positive result)	55,925 (37.1)	19,787 (60.4)	36,138 (30.6)		
	Yes(negative result)	74,108 (49.2)	8989 (27.4)	65,119 (55.2)		

*P* Compared Colonoscopy( +) (%) with Colonoscopy(-) (%), *BMI* body mass index, *Colonoscopy(* +*)* Participants who have complied with colonoscopy, *Colonoscopy(-)* Participants who didn’t comply with colonoscopy

### Outcome ascertainment and quality control

The threshold value for FIT is 100 ng/ml. 4% of stool specimens were chosen at random for retesting as part of quality control. We had all colonoscopies performed by professional gastroenterologists (attending physicians with a minimum of five years' experience in endoscopic operations) in tertiary care hospitals, thereby assuring the quality of diagnostic results. During colonoscopy, examination was performed from the anal canal to the ileocecal region, and all suspicious lesions found during the period were recorded photographically. Data systems recorded all diagnostic information, including morphological features, size (distance from the anus), and location. All screening-related tests were carried out at the CRC screening unit designated by the Tianjin Municipal Health Commission. The HRFQ, FIT, and colonoscopy mentioned above were provided free of charge to all participants. We classified the major colonoscopy findings in the study into four categories: CRC, AA, non-advanced adenoma (NAA), or other benign lesions. CRC included early-stage cancer (T1-2N0M0) and intermediate-stage cancer (all other stages excluding T1-2N0M0). AA was defined as adenomatous polyps ≥ 1 cm in diameter, polyps with a villi component, or high-grade intraepithelial neoplasia. Other benign lesions included non-adenomatous benign lesions as well as benign tumours. To ensure the accuracy in the analysis about the diagnostic yield of colonoscopy, people with poor bowel cleanliness and no clear pathological diagnosis were excluded. In this study, the portion from caecum to splenic flexure of the colon was defined as the proximal colon; it consisted of the caecum, ascending colon, hepatic flexure, transverse colon, and splenic flexure. The portion from the descending colon to the rectum was defined as the distal colon/rectum, which encompassed the descending colon, sigmoid colon, anorectal junction, and rectum.

### Statistical analysis

Colonoscopy data were integrated and analysed to assess the number and distribution of risk factors among participants who underwent colonoscopy, the number and frequency of each lesion detected by colonoscopy, the annual detection rate of CRC and AA, and the anatomical distributions of CRC and AA with NAA found by colonoscopy, as derived from this screening. Categorical variables are described by frequency composition ratios. The correlation of each potential factor with adherence to colonoscopy and the correlation of each risk factor with AA were quantified by odds ratios (ORs), regression coefficients and associated 95% confidence intervals (CIs) using logistic regression models. All statistical analyses were performed using R 4.2.2 statistical software. Statistics were calculated using two-sided tests with a p value of 0.05 or less considered significant.

## Results

### Characteristics of the study population

As shown in Fig. 1, the CRC screening in Tianjin enrolled 5,670,924 participants between 2012 and 2019. After limiting the age range to 40–75 years, a total of 5,226,854 people participated in the HRFQ. As shown in Table 1, a higher proportion of individuals aged between 49 and 69 (71.5%) were included in the study overall. Participants underwent the FIT are dominant (86.3%), and 37.1% (n = 55,925) had positive FIT results. Among the participants who complied with colonoscopy in the high-risk group, 95.5% (n = 31,289) of them were married and 10.7% (n = 29,243) previously detected colonic polyp. Women accounted for 60% (n = 70,753) of the participants who did not comply with colonoscopy, and 57.2% (n = 67,414) performed physical activity less frequently than once a week.

### Colonoscopy compliance and associated factors

In this mass screening, 275,708 high-risk individuals were identified, and 74,685 underwent colonoscopy as recommended, with a compliance rate of 27.1%. The ORs of potentially relevant factors stratified by multiple logistic regression models are presented in Table 2. After adjusting for factors including age, body mass index (BMI), smoking status, and exercise frequency, we found that age, educational background, exercise frequency, a history of chronic diarrhea, a history of chronic constipation, a history of mucous-bloody stools, a history of appendicitis or appendectomy, and a history of gallbladder disease or gallbladder surgery were associated with colonoscopy compliance. For example, participants with a history of mucous bloody stool were 6.99 times more likely to undergo the recommended colonoscopy than participants without a history of mucous bloody stool (OR: 7.99, 95% CI: 7.71–8.28). Participants with a history of chronic diarrhea were 4.78 times more likely to undergo the recommended colonoscopy than those without a history of chronic diarrhea (OR: 5.78, 95% CI: 5.62–5.95). The regression coefficients (95% CI) for participants with a history of mucous and bloody stool, chronic diarrhea, and chronic constipation compared to those in the control group were 2.01 (2.04,2.11), 1.76 (1.73,1.78), and 1.21 (1.18,1.24), respectively. In addition, participants who were highly educated were more willing to undergo a colonoscopy.

**Table 2:** ORs and coefficients of factors associated with colonoscopy compliance in the screening programme

**Risk factors**	**Coefficient**	**(95%CI)**	**Adjusted OR** **(95%CI)**	***P***
**Age(years)**				
40–49	Reference		Reference	
49–59	0.71	(0.66,0.76)	2.03 (1.93,2.13)	< 0.001
59–69	1.16	(1.12,1.21)	3.21 (3.06,3.36)	< 0.001
69–75	1.03	(0.98,1.09)	2.81 (2.67,2.96)	< 0.001
**Sex**				
Women	Reference		Reference	
Men	0.01	(-0.01,0.04)	1.01 (0.99,1.04)	< 0.001
**Educational background**				
Low	Reference		Reference	
Intermediate	0.34	(0.31,0.36)	1.4 (1.37,1.44)	< 0.001
High	0.62	(0.59,0.66)	1.87 (1.80,1.93)	< 0.001
**Alcohol intake**				
Never or occasional	Reference		Reference	
Weekly or daily	0.13	(0.10,0.17)	1.14 (1.10,1.18)	< 0.001
**Frequency of exercise**				
< 1time/week	Reference		Reference	
> 1time/week	0.14	(0.12,0.16)	1.15 (1.13,1.17)	< 0.001
**History of chronic diarrhea**				
No	Reference		Reference	
Y es	1.76	(1.73,1.78)	5.78 (5.62,5.95)	< 0.001
**History of chronic constipation**				
No	Reference		Reference	
Yes	1.21	(1.18,1.24)	3.36 (3.27,3.45)	< 0.001
**History of mucous bloody stool**				
No	Reference		Reference	
Yes	2.01	(2.04,2.11)	7.99 (7.71,8.28)	< 0.001
**History of appendicitis or appendectomy**				
No	Reference		Reference	
Yes	0.87	(0.83,0.91)	2.38 (2.29,2.48)	< 0.001
**History of gallbladder disease or gallbladder surgery**				
No	Reference		Reference	
Yes	0.85	(0.81,0.89)	2.34 (2.24,2.44)	< 0.001

*95% CI* 95% confidence interval, *OR* odds ratio

### Distribution and number of risk factors among participants who underwent colonoscopy

**Fig. 2:**
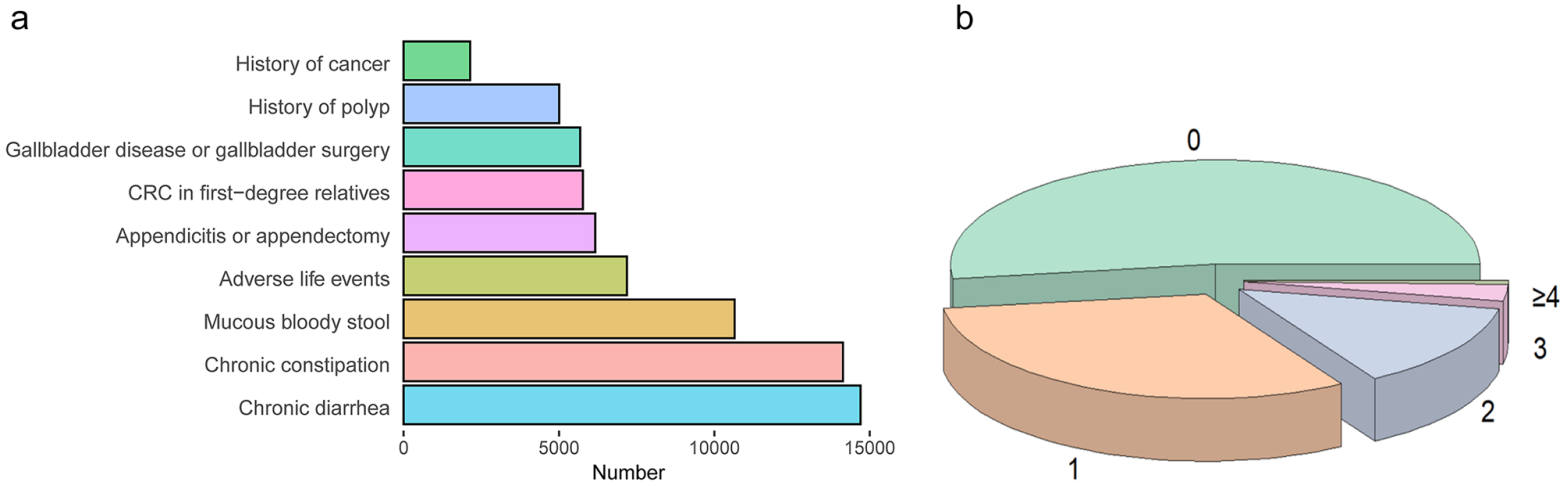
**a** Distribution of risk factors among participants who underwent colonoscopy Colours indicate different risk factors, e.g., blue represents chronic diarrhea. **b** Distribution of the number of risk factors in participants who underwent colonoscopy. Colours indicate different numbers of risk factors; e.g., green represents 0 risk factor

Our analysis of HRFQ data from participants who underwent colonoscopy showed that this population was deeply affected by chronic diarrhea, chronic constipation, mucous bloody stool, and adverse life events (Fig. 2a). Of the 74,685 participants who underwent colonoscopy, 14,703 (19.6%) reported symptoms of chronic diarrhea, and 14,139 (18.9%) reported symptoms of chronic constipation. Individuals who reported symptoms of chronic diarrhea or chronic constipation accounted for two-fifths of all participants who underwent colonoscopy. Additionally, 10,652 participants reported mucous bloody stool, and 7,185 reported a history of adverse life events. These factors ranked third and fourth, respectively.

After compiling the data, we counted the number of HRFQ risk factors reported by each participant who underwent colonoscopy. The distribution of risk factors among participants who underwent colonoscopy is shown in Fig. 2b. The vast majority of these participants (97%) reported no more than three risk factors. More than half participants reported no risk factors.

### Diagnostic yield of colonoscopy

The diagnostic yield of colonoscopy in our screening programme is presented in Table 3. Overall, 1,101 cases of CRC, 4,143 cases of AA, 27,643 cases of NAA, and 7,480 cases of other benign lesions were diagnosed by colonoscopy. The detection rates of CRC, AA, NAA, and other benign lesions were 1.47%, 5.55%, 37.01%, and 10.02%, respectively.

**Table 3:** Lesions detected by colonoscopy in the screening programme

**Findings**	**Participants taking** **screening colonoscopy**	**Detection rates**
**Colorectal cancer**	1101	1.47%
**Advanced adenoma**	4143	5.55%
**Non-advanced adenoma**	27,643	37.01%
**Other benign lesions**	7480	10.02%

**Fig. 3:**
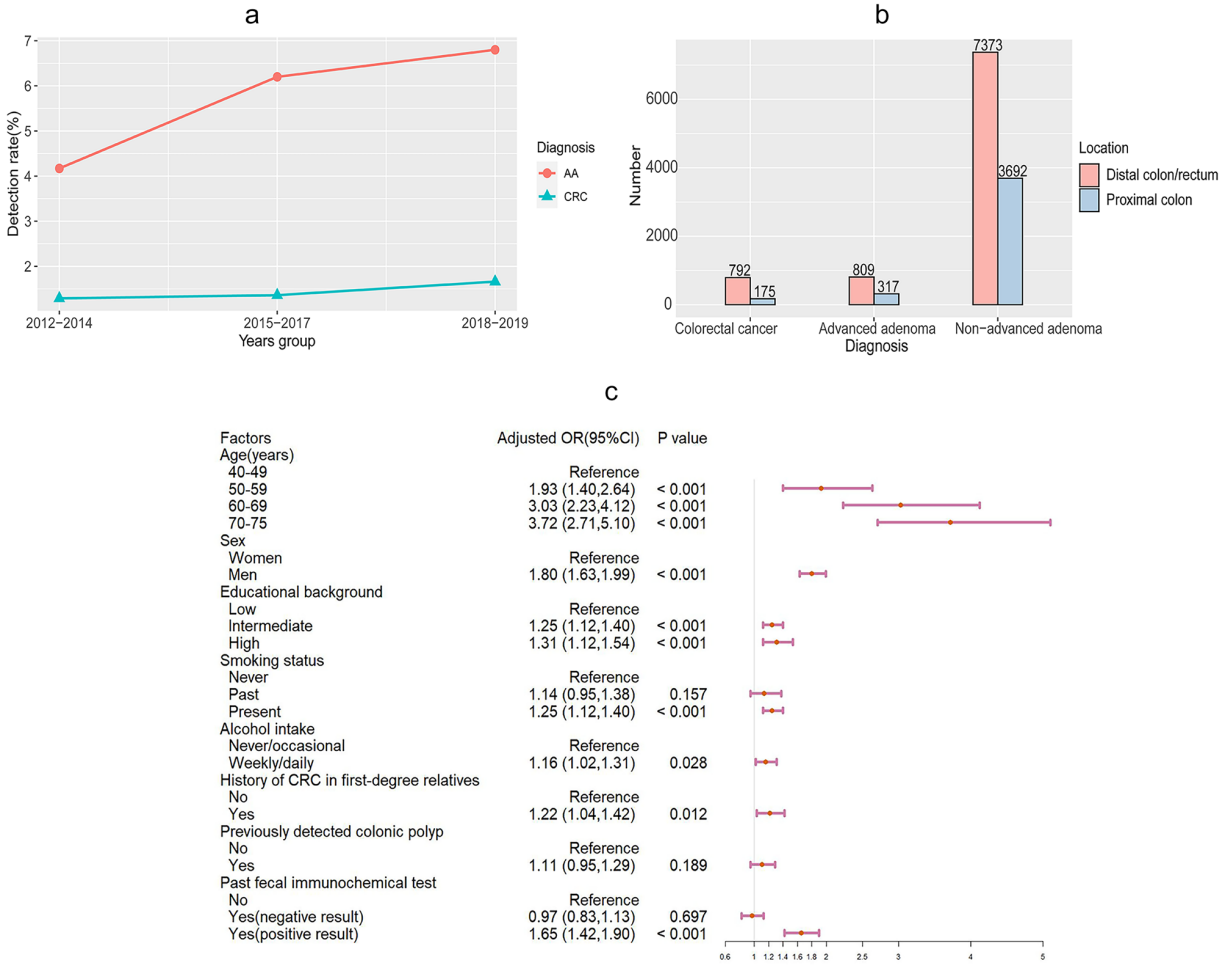
**a** Detection rates of advanced adenoma and colorectal cancer stratified by year CRC: colorectal cancer; AA: advanced adenoma. **b** Anatomical location of advanced adenomas, colorectal cancers and non-advanced adenomas detected by colonoscopy in the screening programme c ORs of risk factors associated with advanced adenoma Analyses were adjusted for age, sex, body mass index (BMI), history of CRC in first-degree relatives, history of colonic polyps, fecal immunochemical test results, exercise frequency, smoking status, alcohol intake and educational background

The detection rates of CRC and AA have gradually increased in recent years. Figure 3a shows the annual changes in the detection rates of the above two lesions. Notably, there was a higher increase in the detection rate of AA than in CRC, from 4.17% (2012–2014) to 6.80% (2018–2019), which is equivalent to 63.10% of the original detection rate.

As far as the anatomical location of CRC, AA, and NAA is concerned, more tumours were detected in the distal colon/rectum than in the proximal colon. In particular, the number of CRC cases detected in the distal colon/rectum (n = 792) was close to four times that detected in the proximal colon. Moreover, 71.8% (n = 809) of AA and 66.6% (n = 7373) of NAA were detected in the distal colon/rectum. The anatomical distribution of the three types of colorectal tumours is shown in Fig. 3b.

### Factors associated with AA

Further, a multiple logistic regression analysis was performed to explore potential risk factors for AA. Figure 3c illustrates the results. Older age, being man, and positive FIT results were significantly and positively associated with AA. For example, the ORs (95% CIs) of AA patients aged 49–59, 59–69, and 69–75 years were 1.93 (1.4–2.64), 3.03 (2.23–4.12), and 3.72 (2.71–5.1), respectively, compared to individuals aged 40–49 years. In addition, being highly educated, smoking, regularly consuming alcohol, and having a history of CRC in first-degree relatives were risk factors for AA. The regression coefficients (95% CI) for men, those with a history of CRC in first-degree relatives, and daily drinkers were 0.59 (0.49, 0.89), 0.2 (0.04, 0.35), and 0.14 (0.01, 0.27) respectively compared to the control group. Regression coefficients for all risk factors are shown in Supplementary Table 1. In our study, other factors, such as exercise frequency, were not associated with AA. The risk factors associated with AA included in this study for analysis are shown in Supplementary Table 2, with references to support them.

## Discussion

Among the 150,676 participants, they were mostly married, older, and had positive FIT results. The likelihood of undergoing a colonoscopy is influenced by a number of variables, with a history of chronic diarrhea, a history of mucous bloody stool, and educational background having the most effects. The HRFQ reported by participants who underwent colonoscopy revealed that the history of adverse life events had a comparatively high rating among risk factors. 83.57% of colonoscopy adherents reported no more than two risk factors in the HRFQ. Factors including age, alcohol intake and smoking status were found to be associated with AA.

Our findings indicate that 95.5% (n = 31,289) of participants who complied with colonoscopy out of 32,762 participants were married, the result consistent with previous studies [21]. After the marriage, people are more likely to take responsibility for their spouses and children, as well as receive more emotional support from their families [22]. This enhances their motivation to comply with the colonoscopy. The colonoscopy compliance rate in our screening programme was 27.1%, which is less than desirable. Compared with Chinese cities without subsidies, our results are lower than those of Shanghai (39.8%) [23] but higher than those of Lin et al.’s screening programme in Guangzhou (18.9%) [20].

We found that age, educational background, exercise frequency were associated with colonoscopy compliance. This study reported stronger positive correlations than those of previous studies, which had a smaller sample size. The logistic regression model in Li et al*.'s* study demonstrated that people with higher educational background (OR: 1.75, 95% CI: 1.11–2.74) and a history of chronic diarrhea (OR: 1.34, 95% CI: 1.00–1.78) were more likely to undergo colonoscopy [24]. People aged 60–64 years (OR: 1.28, 95% CI: 1.21–1.35) and 65–69 years (OR: 1.18, 95% CI: 1.11–1.26) were more compliant with colonoscopy in the study by Chen et al. [25].

These three factors (history of chronic diarrhea, history of chronic constipation, and history of mucous bloody stool) are extremely easy to detect. Among participants who complied with colonoscopy, more than half participants suffered from prolonged these three symptoms. Suspicion of tumour was higher in participants with a history of mucous bloody stool. Thus, they demonstrate a strong motivation to seek medical intervention. In addition, chronic diarrhea should be differentiated from irritable bowel syndrome and ulcerative colitis. A colonoscopy is strongly recommended for participants with such symptoms, who require accurate diagnosis and differentiation.

Furthermore, the educational background of the participants was positively correlated with their colonoscopy compliance. On the one hand, people with greater educational attainment may acquire more health-related knowledge and can thus better understand doctor recommendations; on the other hand, they tend to have higher salaries, such that the cost of a colonoscopy does not bring financial burden. Among adherents to colonoscopy, 7,185 reported adverse life events, a risk factor that ranked fourth on the list. One explanation may be that the loss of some financial and emotional support after a major shock, such as the death of family members or the layoff of adult offspring. Therefore, the free screenings offered and guidance or advice from doctors was appealing. This population needs more care and support in future screenings. Over half of this population without risk factor, which indirectly confirms that the majority of colonoscopy compliance was driven by positive FIT results. Promoting non-invasive stool tests or blood tests and improving test accuracy are also available ways to enhance screening efficiency [26].

We found that the detection rates of CRC, AA, NAA, and other benign lesions among the 74,685 colposcopies were 1.47%, 5.55%, 37.01%, and 10.02%, respectively. Our screening yielded better results than previous studies [27]. These results may be due to the following reasons. In our screening, the doctors performing colonoscopies were well trained and experienced. Furthermore, participants with poor bowel cleanliness or lack of a clear pathological diagnosis were excluded from analysis in our study to prevent impacts on the accuracy of the analysis. The detection rates of CRC and AA increased in recent years, with a more prominent increase in AA detection. Such results are consistent with the conclusion that the incidence of colorectal tumours has increased in recent years. It was found that a substantial number of tumours were detected in the distal colon/rectum. The results of this study are consistent with previous studies [25].

The diagnostic yield of colonoscopy revealed significant results for AA in three aspects: overall detection, detection rates in different years, and detection in the distal colon/rectum. Alcohol can adversely impact tumor immune surveillance, alter DNA repair, change the composition of bile acids, and activate liver cytochrome P-450 enzymes, which can then activate other carcinogens [28].

According to our study, one of its main strengths is that we analyzed the number and distribution of risk factors among participants who have complied with a colonoscopy, which has been less well researched. In addition, our study is based on a large CRC screening programme of 5,670,924 participants, one of the largest datasets available. The large sample size can reduce bias in the results to a certain extent and make them more representative. Besides, we collected a considerable amount of detailed patient information, including epidemiological and clinical-examination data (colonoscopy and pathology). Furthermore, we focused on the rigor of the screening process, all processes were conducted by trained professionals, ensuring the quality of the data.

Yet, there are several aspects that merit caution when interpreting these results. First, the data collection method for the risk factors was self-reported by the participants who volunteered to take part in the screening. Some people is also at risk, but because they do not wish to participate, we are unable to collect their data, and the self-reporting method is undoubtedly subjective. Second, our sample consisted of individuals only from Tianjin; thus, there are limitations to its representativeness and the ability to generalize the results to other regions. More regions and health centres will be included in our future studies. Third, the limited data collected in the present study did not address other relevant factors (e.g., the type and coverage of health insurance, regularity of medical check-ups) associated with colonoscopy compliance.

The colonoscopy compliance, a key component of CRC screening, urgently needs to be improved. Particular attention should be paid to those with low educational background. Besides, we need show solicitude for participants with a history of adverse life events in future screenings and promote participation in non-invasive tests. Recently, evidence has grown to support the idea that serrated polyps (SPs) are another precursor lesion for CRC [29]. The sporadic MSI and the CpG island methylation phenotype (CIMP), the latter of which is believed to represent the key mechanism of CRC induced by serrated lesions, are two different molecular pathways [30]. This aspect should be given attention in future screenings. The results of this study can inform future population-based CRC screening programmes.

### Supplementary Information

Below is the link to the electronic supplementary material.

Supplementary file1 (DOCX 43 KB)

## Data Availability

The datasets analysed during the current study are not publicly available due to limitations of ethical approval involving the patient data and anonymity but are available from the corresponding author on reasonable request.

## Abbreviations

CRC: Colorectal cancer
AA: Advanced adenoma
NAA: Non-advanced adenoma
FIT: Fecal immunochemical tests
HRFQ: High-risk factor questionnaire
95% CI: 95% Confidence interval
OR: Odds ratios
BMI: Body mass index
SPs: Serrated polyps
CIMP: CpG island methylation phenotype
