# Supplementary material for: Colonoscopy compliance and diagnostic yield in a large population-based colorectal cancer screening programme
Source: Int J Colorectal Dis. 2023 Sep 13;38(1):227. doi: 10.1007/s00384-023-04517-3 (PMC10497689; doi:10.1007/s00384-023-04517-3)
Supplement: Supplementary file 1 — Supplementary file1 (DOCX 43 KB) [file 384_2023_4517_MOESM1_ESM.docx]

| **Supplementary Table 1 ORs and coefficients of risk factors associated with advanced adenoma.** | | | | |
| --- | --- | --- | --- | --- |
| **Factors** | **Coefficient** | **(95%CI)** | **OR(95%CI)** | ***P*** |
| **Age(years)** |  |  |  |  |
| 40-49 |  |  | Reference |  |
| 50-59 | 0.66 | (0.35,0.99) | 1.93 (1.40,2.64) | < 0.001 |
| 60-69 | 1.11 | (0.81,1.43) | 3.03 (2.23,4.12) | < 0.001 |
| 70-75 | 1.31 | (1.01,1.64) | 3.72 (2.71,5.10) | < 0.001 |
| **Sex** |  |  |  |  |
| Women |  |  | Reference |  |
| Men | 0.59 | (0.49,0.89） | 1.80 (1.63,1.99) | < 0.001 |
| **Educational background** |  |  |  |  |
| Low |  |  | Reference |  |
| Intermediate | 0.22 | (0.11,0.37) | 1.25 (1.12,1.40) | < 0.001 |
| High | 0.27 | (0.11,0.49) | 1.31 (1.12,1.54) | < 0.001 |
| **Smoking status** |  |  |  |  |
| Never |  |  | Reference |  |
| Past | 0.13 | (-0.06,0.32) | 1.14 (0.95,1.38) | 0.157 |
| Present | 0.23 | (0.11,0.34) | 1.25 (1.12,1.40) | < 0.001 |
| **Alcohol intake** |  |  |  |  |
| Never/occasional |  |  | Reference |  |
| Weekly/daily | 0.14 | (0.01,0.27) | 1.16 (1.02,1.31) | 0.028 |
| **History of CRC in first-degree relatives** |  |  |  |  |
| No |  |  | Reference |  |
| Yes | 0.20 | (0.04,0.35) | 1.22 (1.04,1.42) | 0.012 |
| **Previously detected colonic polyp** |  |  |  |  |
| No |  |  | Reference |  |
| Yes | 0.10 | (-0.05,0.25) | 1.11 (0.95,1.29) | 0.189 |
| **Past fecal immunochemical test** |  |  |  |  |
| No |  |  | Reference |  |
| Yes(negative result) | -0.03 | (-0.18,0.13) | 0.97 (0.83,1.13) | 0.697 |
| Yes(positive result) | 0.50 | (0.35,0.65) | 1.65 (1.42,1.90) | < 0.001 |
| Analyses were adjusted for age, sex, body mass index (BMI), history of CRC in first-degree relatives, history of colonic polyps, fecal immunochemical test results, exercise frequency, smoking status, alcohol intake and educational background.  Abbreviations: 95% CI, 95% confidence interval; OR, odds ratio. | | | | |

| **Supplementary Table 2 Risk factors associated with advanced adenoma.** |
| --- |
| **Risk factors** **References** |
| \| Sex (1, 2) \| \| --- \| \| Age (3, 4) \| \| BMI (5) \|  \| \| Smoking status (6) \|  \| \| Alcohol intake (7, 8) \|  \| \| Educational background (9) \| \| \| Exercise frequency (10, 11) \|  \| \| History of CRC in first-degree relatives (12, 13) \| \| \| History of colonic polyps (14) \| \| \| Past fecal immunochemical test (15, 16) \|  \| |

1. Ferlitsch M, Reinhart K, Pramhas S, Wiener C, Gal O, Bannert C, et al. Sex-specific prevalence of adenomas, advanced adenomas, and colorectal cancer in individuals undergoing screening colonoscopy. Jama. 2011;306(12):1352-8.

2. Kim JY, Jung YS, Park JH, Kim HJ, Cho YK, Sohn CI, et al. Different risk factors for advanced colorectal neoplasm in young adults. World journal of gastroenterology. 2016;22(13):3611-20.

3. Strul H, Kariv R, Leshno M, Halak A, Jakubowicz M, Santo M, et al. The prevalence rate and anatomic location of colorectal adenoma and cancer detected by colonoscopy in average-risk individuals aged 40-80 years. Am J Gastroenterol. 2006;101(2):255-62.

4. de Jonge V, Sint Nicolaas J, van Leerdam ME, Kuipers EJ, Veldhuyzen van Zanten SJ. Systematic literature review and pooled analyses of risk factors for finding adenomas at surveillance colonoscopy. Endoscopy. 2011;43(7):560-72.

5. Kim SE, Shim KN, Jung SA, Yoo K, Moon IH. An association between obesity and the prevalence of colonic adenoma according to age and gender. Journal of gastroenterology. 2007;42(8):616-23.

6. Lieberman DA, Prindiville S, Weiss DG, Willett W. Risk factors for advanced colonic neoplasia and hyperplastic polyps in asymptomatic individuals. Jama. 2003;290(22):2959-67.

7. Čebohin M, Samardžić S, Marjanović K, Tot Vesić M, Kralik K, Bartulić A, et al. Adenoma Characteristics and the Influence of Alcohol and Cigarette Consumption on the Development of Advanced Colorectal Adenomas. International journal of environmental research and public health. 2020;17(22).

8. Shin A, Hong CW, Sohn DK, Chang Kim B, Han KS, Chang HJ, et al. Associations of cigarette smoking and alcohol consumption with advanced or multiple colorectal adenoma risks: a colonoscopy-based case-control study in Korea. American journal of epidemiology. 2011;174(5):552-62.

9. Burnett-Hartman AN, Passarelli MN, Adams SV, Upton MP, Zhu LC, Potter JD, et al. Differences in epidemiologic risk factors for colorectal adenomas and serrated polyps by lesion severity and anatomical site. American journal of epidemiology. 2013;177(7):625-37.

10. Rezende LFM, Lee DH, Keum N, Nimptsch K, Song M, Lee IM, et al. Physical activity during adolescence and risk of colorectal adenoma later in life: results from the Nurses' Health Study II. Br J Cancer. 2019;121(1):86-94.

11. Wallace K, Baron JA, Karagas MR, Cole BF, Byers T, Beach MA, et al. The association of physical activity and body mass index with the risk of large bowel polyps. Cancer epidemiology, biomarkers & prevention : a publication of the American Association for Cancer Research, cosponsored by the American Society of Preventive Oncology. 2005;14(9):2082-6.

12. Gupta A, Samadder J, Elliott E, Sethi S, Schoenfeld P. Prevalence of adenomas and advanced adenomas in patients in the 40- to 49-year age group undergoing screening colonoscopy because of a family history of adenoma/polyp in a first-degree relative. Gastrointestinal endoscopy. 2012;75(4):705-11.

13. Samadder NJ, Curtin K, Tuohy TM, Rowe KG, Mineau GP, Smith KR, et al. Increased risk of colorectal neoplasia among family members of patients with colorectal cancer: a population-based study in Utah. Gastroenterology. 2014;147(4):814-21.e5; quiz e15-6.

14. Saini SD, Kim HM, Schoenfeld P. Incidence of advanced adenomas at surveillance colonoscopy in patients with a personal history of colon adenomas: a meta-analysis and systematic review. Gastrointestinal endoscopy. 2006;64(4):614-26.

15. Yeh JH, Lin CW, Wang WL, Lee CT, Chen JC, Hsu CC, et al. Positive Fecal Immunochemical Test Strongly Predicts Adenomas in Younger Adults With Fatty Liver and Metabolic Syndrome. Clinical and translational gastroenterology. 2021;12(2):e00305.

16. Kligman E, Li W, Eckert GJ, Kahi C. Adenoma Detection Rate in Asymptomatic Patients with Positive Fecal Immunochemical Tests. Digestive Diseases and Sciences. 2018;63(5):1167-72.
